# Coordination and Consonance Between Interacting, Improvising Musicians

**DOI:** 10.1162/opmi_a_00036

**Published:** 2020-11-01

**Authors:** Matthew Setzler, Robert Goldstone

**Affiliations:** Program in Cognitive Science, Indiana University; Program in Cognitive Science, Indiana University; Department of Psychological and Brain Sciences, Indiana University

**Keywords:** joint action, distributed cognition, improvisation, time series modeling, music

## Abstract

Joint action (JA) is ubiquitous in our cognitive lives. From basketball teams to teams of surgeons, humans often coordinate with one another to achieve some common goal. Idealized laboratory studies of group behavior have begun to elucidate basic JA mechanisms, but little is understood about how these mechanisms scale up in more sophisticated and open-ended JA that occurs in the wild. We address this gap by examining coordination in a paragon domain for creative joint expression: improvising jazz musicians. Coordination in jazz music subserves an aesthetic goal: the generation of a collective musical expression comprising coherent, highly nuanced musical structure (e.g., rhythm, harmony). In our study, dyads of professional jazz pianists improvised in a “coupled,” mutually adaptive condition, and an “overdubbed” condition that precluded mutual adaptation, as occurs in common studio recording practices. Using a model of musical tonality, we quantify the flow of rhythmic and harmonic information between musicians as a function of interaction condition. Our analyses show that mutually adapting dyads achieve greater temporal alignment and produce more consonant harmonies. These musical signatures of coordination were preferred by independent improvisers and naive listeners, who gave higher quality ratings to coupled interactions despite being blind to condition. We present these results and discuss their implications for music technology and JA research more generally.

## INTRODUCTION

High-level cognition is often achieved by groups of interacting individuals (Knoblich et al., 2011; Sebanz et al., 2006). Group behavior in joint action (JA) settings is less dependent on isolated individual efforts and more on the ability to coordinate (Goldstone & Gureckis, 2009; Hasson et al., 2012). Insight into the mechanisms underlying successful coordination has important implications for how we understand interpersonal interaction, optimize team performance, and engineer humanlike artificial intelligence systems (Cooke & Hilton, 2015; Guimera et al., 2005; Rebsamen et al., 2010; D. C. Richardson et al., 2007). This study examines coordination in collaboratively improvising jazz musicians. Coordination in jazz music subserves an aesthetic goal: the generation of a collective musical expression, and the expertise of professional jazz musicians lies largely in their ability to coordinate and adapt spontaneously in real-time performance. Professional jazz ensembles thus offer a remarkably sophisticated paragon domain to study the basic properties and limits of our capacity to coordinate with one another.

Humans align their behaviors as they interact (Hasson & Frith, 2016; Pickering & Garrod, 2004, 2013). We spontaneously entrain periodic motions (e.g., postural sway, walking gait), and such entrainment is predictive of successful interaction and performance on joint tasks (Demos et al., 2012; Paxton & Dale, 2013; M. J. Richardson et al., 2007; Schmidt & Richardson, 2008; Shockley et al., 2003; Shockley et al., 2009; Valdesolo et al., 2010). Interlocutors tend to mirror one another’s posture, speech prosody and align eye gaze to fixate on the same objects as they interact (Garrod & Pickering, 2009; Louwerse et al., 2012; D. C. Richardson & Dale, 2005; D. C. Richardson et al., 2007; D. C. Richardson et al., 2009). Alignment occurs at more abstract levels as well. Interlocutors mirror vocabulary and syntactical constructions, and come to share common mental representations for situations under discussion (Abney et al., 2014; Dale & Spivey, 2006; Pickering & Garrod, 2004). Past JA research demonstrates that alignment is an important interpersonal mechanism that facilitates joint attention and predictive emulation (of a partner’s future actions), and streamlines communication by providing a common representational scheme (Garrod & Pickering, 2009; Pickering & Garrod, 2004; D. C. Richardson et al., 2009; Sebanz et al., 2006; Sebanz & Knoblich, 2009).

Another issue in JA research is whether group behavior is supported by mutual adaptations (bidirectional coordination) or fixed leader-follower roles (unidirectional coordination). Clearly delineated leader-follower roles appear to support stable coordination in many naturalistic JA domains (e.g., conductor of an orchestra, lead dancer in a salsa pair), and experimental studies have affirmed the utility of unidirectional coordination with respect to particular task constraints and participant expertise levels (Curioni et al., 2019; Noy et al., 2011; M. J. Richardson et al., 2015). On the other hand, finger-tapping studies have shown that dyads achieve greater synchronization when mutually coupled compared to unidirectional conditions (Demos et al., 2017; Konvalinka et al., 2010). Rather than adopting leader-follower roles, mutually coupled individuals each adapted their own tapping rates to their partner’s previous tapping rates (Konvalinka et al., 2010). A similar result has been observed in a simplified experimental adaptation of the “mirror game,” which requires dyads to synchronize improvised hand movements with one another. Mutually coupled dyads synchronized more fluidly and generated more dynamic movements compared to dyads that were assigned leader-follower roles (Noy et al., 2011).

These findings show that mutual coupling often promotes coordination by supporting robust and flexible behavioral alignment. However, they were obtained in idealized experimental paradigms using greatly simplified behaviors (e.g., synchrony of a tapped pulse), so it is unclear whether and how they generalize to more sophisticated coordinated behavior found in the real world. Naturalistic JA is often open-ended, and requires not just behavioral matching but also *complementary* coordination in service of abstract, functional goals (e.g., operating on a patient, generating ideas in group brainstorming sessions; Hasson & Frith, 2016; Paulus et al., 2010). How does mutual coupling shape coordination in these more complex, naturalistic forms of JA? Does mutual coupling support greater behavioral alignment in underconstrained tasks, where this is no explicit goal of synchronization? Does it support *complementary* coordination, in service of abstract goals?

In this study we use improvised music as a model domain to explore the effects of mutual coupling in the wild. Conveniently, joint music performance is naturally mediated by organizational structures that constrain ensemble coordination. Orchestras are hierarchically organized with fixed leader-follower roles, whereas free improvising jazz ensembles are typically more characterized by feedback loops of mutual influence (Borgo, 2005; D’Ausilio et al., 2012). Studio recording practices such as “overdubbing” also constrain coordination by sequentially recording individual musical parts. Ensemble performance research has shown that these underlying patterns of coordination are reflected in the music and movements of ensemble members (Hennig, 2014; Keller, 2014; Rasch, 1979), such as small temporal asynchronies of co-performer note onsets (Demos et al., 2017; Goebl & Palmer, 2009; Keller & Appel, 2010; Palmer & Zamm, 2017), and postural sway couplings (Chang et al., 2017; Eerola et al., 2018).

Improvised music is of particular interest, because the influence of coordination extends beyond sensorimotor coupling and into the music’s formal architecture, which is freely evolving over time in its rhythm, melody, harmony, and texture. We might thus expect underlying coordination patterns to constrain these structural elements, similar to how they constrain sensorimotor coupling in scored music performance. Do mutually coupled improvisers engage in bidirectional coordination at the level of notes and rhythms? If so, does this result in higher quality music? Answering these questions will extend our understanding of JA beyond idealized laboratory tasks and into sophisticated, open-ended coordination that occurs in elite artistic performances. It will also yield direct implications for music technology. Results will reveal repercussions of the popular recording technique of overdubbing, and our quantitative measures of improvised musical coordination can be incorporated into artificial interactive music systems (Gillick et al., 2019; Linson et al., 2015) and benefit music pedagogy by automating assessment of ensemble performance.

Despite a paucity of cognitive science research on collective improvisation, some notable efforts have begun. Previous studies have shown that improvised musical coordination is shaped by musical context (e.g., playing with a drone versus a swing backing track), and that experimentally manipulated social attitudes (e.g., dominant, caring) are sonically encoded in improvised musical interactions (Aucouturier & Canonne, 2017; Walton et al., 2018). These studies lay an important foundation, but they did not experimentally isolate mutual coupling between musicians. Moreover, their analyses did not incorporate music theory, and thus the findings are limited to temporal and acoustic coordination properties, and do not extend to more abstract musical phenomena such as the emergence of tonal structure (i.e., harmony, melody).

In the current study we directly manipulate interaction in co-improvising musicians, and examine how different underlying patterns of coordination constrain the exchange and emergence of rhythmic and tonal information. Professional jazz musicians freely improvised in two duo conditions: a *coupled* condition, in which both pianists improvised simultaneously, and a *one-way* condition, in which a single pianist improvised along with a recording of another pianist (a “ghost partner”) from a previous *coupled* duet. Improvisations were completely “free” in the sense that there was no predetermined songform, key signature, or tempo; the only instruction was to improvise a compelling piece of music de novo, as in an actual performance. These duo conditions provided two naturalistic musical settings to isolate the effects of mutual coupling in freely improvising musicians. Whereas *coupled* duos had the ability to mutually adapt to one another, *one-way* duos were restricted to unidirectional coordination (i.e., because the “ghost partner” was unresponsive to the live musician), as in the common studio recording technique of overdubbing.

Participants were recorded in isolated MIDI^1^ tracks as they improvised in each condition. Time series of two fundamental musical features were extracted and analyzed: onset density and tonal consonance. Onset density indexes overall rhythmic activity level, and has been shown to correlate with listener perception of musical tension (Farbood, 2012). Tonal consonance refers to how different combinations of notes sound on a continuum from dissonant/unstable to consonant/stable (Johnson-Laird et al., 2012), and was operationalized using a previously established model of musical tonality, the tonal spiral array (Chew, 2005, 2014; Herremans & Chew, 2016). We find that interaction condition systematically altered the coordinated musical behavior of dyads, who were more rhythmically coupled and produced more consonant tonal structure when mutually coupled. These effects were paralleled in the subjective experiences of participants as well as nonmusician listeners, who preferred *coupled* duets despite being blind to condition. These results are presented and discussed in terms of their implications for music technology and JA research more generally.

## METHODS

### Participants

Twenty-eight professional pianists (25 male, 3 female) from the New York City jazz scene participated in this study. Participant age ranged from 21 to 37. On average participants had over 22 years’ experience playing piano (*SD* = 5.2) and 15 years experience improvising (*SD* = 4.6). All participants had extensive experience with free improvisation, and received formal training in piano performance and/or jazz studies at elite conservatories. Participants were recruited by word of mouth, and had no prior experience performing with one another.

There were 122 individuals that participated in the listener study. Of those, 101 were undergraduate psychology students from Indiana University without any particular musical background, and 21 (19 male, 2 female) were professional jazz musicians, each with over 10 years of experience as improvising musicians, recruited by word of mouth from the New York City music scene. None of these listeners participated in the initial music-generation stage of the study.

### Design and Procedure

Participants played a series of short (4–7 min “free”) improvisations, with no accompanying stimuli and no prior musical template or constraints. Other than the suggested time frame, the only instruction was to improvise a compelling piece of music, as in a typical performance setting. Participants were informed of the two interaction conditions, but were not told which condition they were playing in on any given trial (and there was no visual or audible indication of condition, see the Supplemental Materials, Setzler & Goldstone, 2020). After each trial, they responded to questionnaires indicating their subjective experience playing in the previous trial in terms of: (1) how easy it was to coordinate with their partner, (2) how well coordinated they were with their partner, (3) quality of the improvised piece, and (4) degree to which they played a leader versus a supporter role.

Each participant played at least three duets (trials) in each condition, with the same “live” partner for every *coupled* duet and the same “ghost” partner for every *one-way* duet. Conditions were interleaved within participant pairs and counterbalanced across pairs to control for possible order effects. Participants were recorded in isolated MIDI tracks, and individual recordings from *coupled* duets yoked *one-way* duets in subsequent sessions, as depicted in the Supplemental Materials (Setzler & Goldstone, 2020). Altogether 50 *coupled* duets and 86 *one-way* duets were collected; duets had an average duration of 342 s (min = 108 s, max = 738 s, *SD* = 12 s). See the Supplemental Materials for a link to the publicly hosted data set (Setzler & Goldstone, 2020).

A post hoc study was conducted with populations of naive listeners and expert jazz musicians. Listeners heard 30-s audio clips randomly sampled from duets in both conditions (audio from each pianist was panned to separate ears). After listening to each clip they were asked to rate (1) their enjoyment of the music, (2) how well coordinated they perceived the musicians to be, and (3) which musician played more of a leader role. Listeners were also asked to guess which condition a clip came from. Each participant heard complementary yoked sets of *coupled* and *one-way* clips. See the Supplemental Materials for full specification of the sequencing design, which controlled for possible order and stereo-panning effects (Setzler & Goldstone, 2020).

### Tonal Consonance Measure

Our tonal consonance measure is based on the tonal spiral array model, which has been validated against listener ratings and expert music theory analyses (Chew, 2005, 2014; Herremans & Chew, 2016). Table 1 shows model ratings for exemplar pitch sets. See the Supplemental Materials for specification of the measure (Setzler & Goldstone, 2020).

**Table 1.** Consonance ratings of exemplar pitch sets.

**Pitch Set**	**Consonance**
{C, E, G} (Cmaj)	.65
{C, Eb, G} (Cmin)	.65
{C, B, G}	.54
{C, E, G, F, A, C} (Cmaj + Fmaj)	.49
{C, B}	.48
{C, E, G, F#, A#, C#} (Cmaj + F#maj)	.13
serial (all 12 pitches)	.09

### Data Analysis

Listener ratings were analyzed with Bayesian mixed-effects models for each response type, using the brms package in R (Bürkner, 2016; Carpenter et al., 2017). Instead of predicting enjoyment and coordination ratings directly, models predicted the difference between ratings of *coupled* audio clips minus ratings of correspondingly yoked *one-way* clips, such that positive intercepts indicated preference for *coupled* clips. Leadership ratings within *one-way* trials were modeled such that positive intercepts indicated perception of “ghosts” leading, and negative values indicated perception of live musicians leading. Accuracy of condition guesses was modeled as binomial outcome: whether or not listeners guessed the correct condition, such that positive intercepts indicated above-chance predictions. Models included a predictor for subject type (naive listener or professional jazz musician), and random intercepts per individual. Bayesian mixed-effects models were also used to analyze time series measures of musical coordination (cross-correlation of onset density and lagged consonance, see Results). Dependent measures were predicted by a fixed-effect of interaction condition, with random intercepts for yoked groupings at the duo and duet levels.^2^ Unidirectional coordination in *one-way* duos was analyzed by predicting dependent measures as a function of lag direction (i.e., ghost-to-live versus live-to-ghost), with random-effects for each duo and duet.

## RESULTS

### Subjective Ratings

Despite being blind to condition, performers and naive listeners both exhibited a strong preference for *coupled* over *one-way* duets. Performers rated *coupled* trials as producing higher quality music (21 out of 26 performers rated coupled higher; probability of success = 0.81; exact binomial test *p* < .01). Coupled trials were also rated as being better coordinated (23 out of 26 performers rated coupled trials as being better coordinated; probability of success = 0.88; binomial test *p* < .01), and more easily coordinated (24 out of 26 performers found it easier to coordinate with their partner on coupled trials; probability 0.92; *p* < .01). Performers also rated themselves as playing more of a supportive (versus lead) role in *one-way* duos, whereas leadership was rated to be more evenly distributed throughout *coupled* duos (difference between average ratings within participant by condition; paired *t*(25) = 3.16, *p* < .01).

Bayesian mixed-effects models predicting the *difference* in listener ratings between *coupled* clips and correspondingly yoked *one-way* clips indicated that listeners found *coupled* clips to be more enjoyable (*M* = .24, *SD* = .08, 95% CI = [.08, .40]) and better coordinated (*M* = .43, *SD* = .11, 95% CI = [.21, .64]). Listeners also perceived unresponsive “ghost partners” to lead live musicians in *one-way* duos (*M* = .14, *SD* = .03, 95% CI = [.08, .20]), whereas leadership was perceived to be more evenly distributed in *coupled* duos (effect of condition on deviation of leadership ratings from neutral: *M* = .14, *SD* = .03, 95% CI = [.08, .19]). However, listeners did not guess the correct condition above chance level (*M* = .03, *SD* = .09, 95% CI = [−.14, .21]). These results held equally for both populations of listeners, as no effects of subject type were observed.

### Mutual Coupling Promotes Synchrony

**Figure 1.**
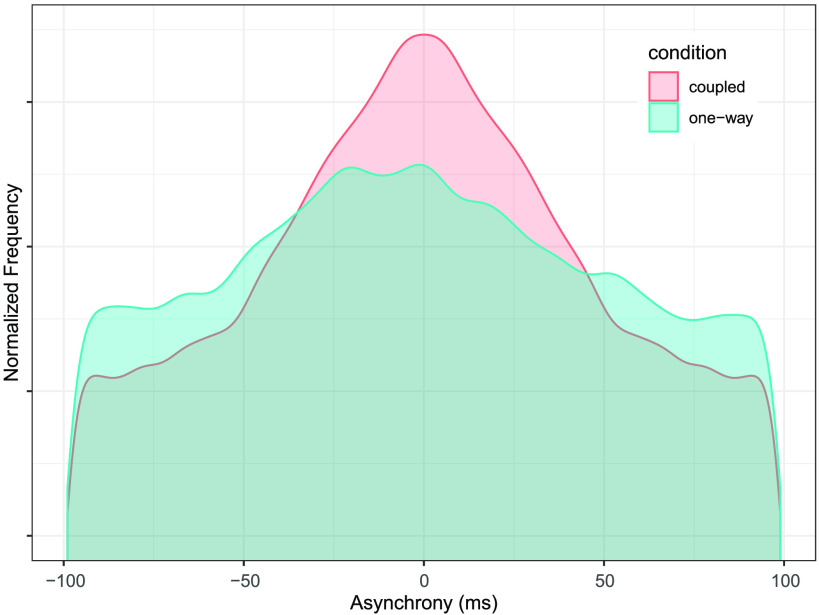
**Mutual coupling facilitates precise synchronization.** Distribution of asynchronies (asyncs) between co-performers’ near-simultaneous (within 100 ms) note onsets throughout all trials in each condition. Asyncs are more tightly clustered around 0 in *coupled* trials, indicating more precise temporal alignment. Asyncs are mathematically symmetric around 0 for *coupled* trials.

How does coupling influence musicians’ ability to synchronize with one another? Asynchronies between “near-simultaneous” onsets (co-occurring within 100 ms) played by co-performers were measured throughout all duets in each condition. Near-zero asynchronies indicate close temporal alignment, while asynchronies of larger magnitude reflect less precise synchronization. As depicted in Figure 1, asynchronies in *coupled* trials peak around zero (red distribution), whereas asynchronies in *one-way* trials are more widely distributed throughout the +/− 100 ms range (blue distribution) (KS.test D = 0.024, *p* < .01), indicating that mutually coupled musicians achieved more precise synchronization compared to musicians in the overdubbed condition. We were also curious about leader-follower asymmetries in *one-way* duos, as previous studies have reported that supporting musicians lag behind lead musicians in certain composed musical contexts (Keller & Appel, 2010) (asynchronies are mathematically symmetric around zero for *coupled* trials because each asynchrony was computed from the perspective of both live musicians, but asynchronies were only computed from perspective of the single live musician in *one-way* trials). However, no such effects were observed here; the distribution of asynchronies in *one-way* duets was not significantly asymmetric around 0 in one direction or the other (mixed-effects model of asynchronies from *one-way* duos, with random-intercepts per duo: intercept = −.54, *SE* = .34, *t*(18.63) = −1.58, *p* = .13; model fit with lme4 package in R, significance assessed with lmerTest using Satterthwaite’s method used to estimate degrees of freedom; Bates et al., 2014; Kuznetsova et al., 2017).

### Activity Matching

**Figure 2.**
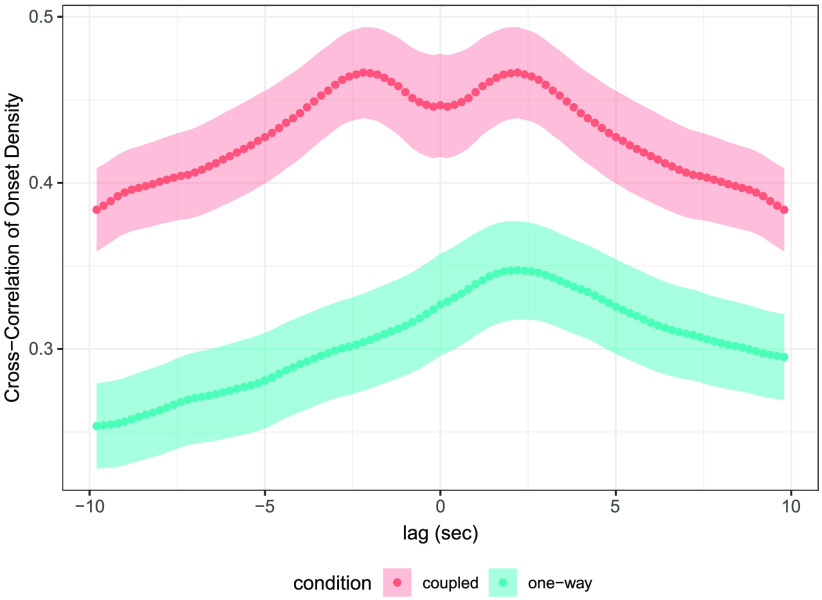
**Musicians match the activity level of their partners.** Points represent mean lagged cross-correlation across all trials within each condition. Error ribbons denote standard error of the mean. Positive lags in *one-way* trials represent correlation of ghost recording onset density with future onset density of live musicians (ghost-to-live) and vice versa for negative lags (live-to-ghost). Cross-correlation is mathematically symmetric around 0 for *coupled* trials.

Lagged cross-correlation of co-performers’ onset density was computed to analyze how musicians responded to one another’s rhythmic activity level. Onset density contributes to the perception of musical tension (Farbood, 2012). A frenzied musical passage comprising many notes in rapid succession would yield high onset density, whereas a more sparse, mellow passage would yield low-onset density. Onset density time series were computed for each individual note sequence using a 2-s sliding window, with a 0.2-s hop size. Figure 2 depicts lagged cross-correlations, averaged across all duets in each condition. Cross-correlation was positive overall (cross-correlation averaged across +/− 20 s lag range: *M* = .39, *SD* = .04, 95% CI = [.31, .47]), but significantly higher in *coupled* duos (red curve) (fixed effect of condition: *M* = −.13, *SD* = .04, 95% CI = [−.21, −.06]). These results indicate a general tendency for musicians to match the onset density of their partners, which was exaggerated in mutually coupled duos.

Within *one-way* duos, cross-correlation was significantly higher at positive, “ghost-to-live” lags (onset density of ghost recording correlated with future onset density of live musician) compared to negative, “live-to-ghost” lags (effect of direction: *M* = .05, *SD* = .01, 95% CI = [.02, .08]). This reflects the underlying asymmetry in *one-way* duos: live musicians were responsive to notes of ghost recordings but not the other way around. (In contrast, cross- is mathematically symmetric around zero for *coupled* trials, because it was computed from the perspective of both musicians in each duo.) As reported in the Supplemental Materials (Setzler & Goldstone, 2020), a complementary Granger Causality analysis also revealed greater ghost-to-live versus live-to-ghost Granger causality in *one-way* duos. Lastly, Figure 2 reveals a dip in cross-correlation for coupled duets at simultaneous timepoints, but this was not statistically significant (paired *t* test of average cross-correlation at lag-0 versus 2-s lag across all trials for each coupled duo; *t*(13) = −1.6335, *p* = .063).

### Emergence and Directed Flow of Tonal Information

A previously established model of tonal structure (see Methods and Supplemental Materials, Setzler & Goldstone, 2020) was adapted to provide a measure of *tonal consonance*, quantifying how collections of notes sound on a continuum from unstable/dissonant to stable/consonant (Chew, 2014; Herremans & Chew, 2016). Time series of combined consonance (consonance of merged music streams from both players in a duo) were computed with a sliding window.^3^ Emergent consonance (EC) was operationalized as Combined consonance minus average consonance of each individual music stream. EC captures the consonance arising from the *interaction* of pitches played by collaborating musicians. A situation in which each pianist plays self-consonant notes that clash with one another would result in low EC (e.g., {C, E, G} and {F#, A#, C#} are consonant on their own but {C, E, G, F#, A#, C#} is highly dissonant), whereas a situation in which each pianist plays dissonant notes that stabilize one another when sounded together would result in high EC (e.g., {C, B} and {E, G} have low average consonance but {C, E, G, B} has high consonance because it is tonicized to a Cmaj7 chord). Negative EC values indicate that combined consonance is less consonant than the average individual consonance and can be interpreted as emergent *dissonance*. Less negative values can be interpreted as indexing greater EC (less emergent dissonance) compared to more negative values.

A novel lagged consonance analysis was conducted to quantify how musicians harmonized with one another’s notes as a function of interaction condition. Lagged consonance was computed by shifting individual note sequences of co-performers relative to one another, computing combined and emergent consonance time series of the merged pitch collections with a sliding window, and then averaging over time to get a single consonance value per piece at each lag (5-s sliding window and 2-s hop size were used, although these results were robust across a range of window sizes, as documented in the Supplemental Materials, Setzler & Goldstone, 2020). This analysis captures the directed flow of tonal information, as it quantifies the degree to which individuals harmonized with the preceding notes of their partner. For example, Player A might harmonize with Player B’s past notes but not the other way around, which would be reflected in high consonance for B-to-A lags but not A-to-B lags. Lagged consonance was computed for every trial in each condition with lags in the range of +/− 20 seconds, spaced by increments of 2 s. Positive lags in *one-way* duos correspond to evaluating past notes of the ghost recording with future notes of the live musician (ghost-to-live) and vice versa for negative lags (live-to-ghost). The beginnings and endings of pieces (first and last 10%) were discarded to avoid boundary effects.

**Figure 3.**
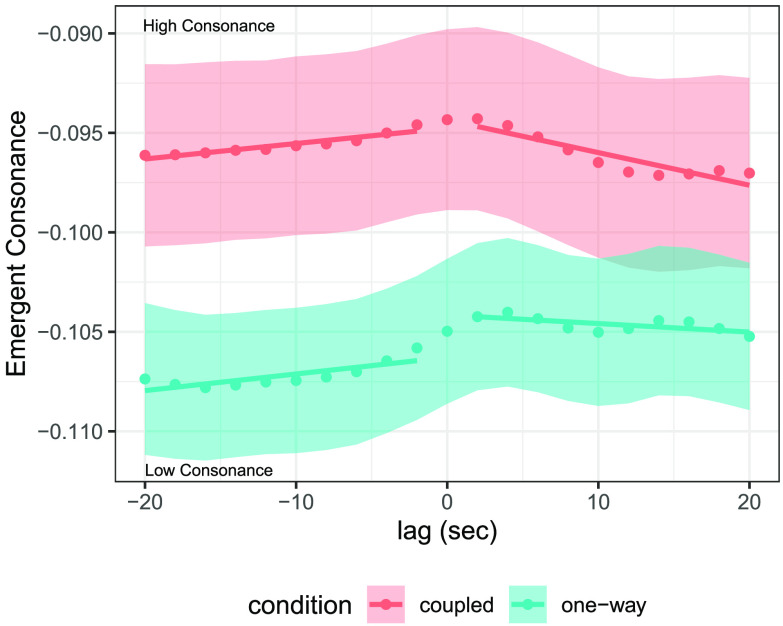
**Lagged consonance analysis reveals musicians harmonize with preceding notes of their partner.** Points denote average emergent consonance (EC) at a given lag across every piece within each condition, error bars denote standard error of the mean. Negative lags correspond to notes of the live musician merged with future notes of the ghost recording (live-to-ghost) and vise versa for positive lags (ghost-to-live). Linear fits of EC by lag are shown for negative and positive lags in each condition.

Figure 3 depicts average lagged EC by condition. Time lags are plotted on the *x*-axis, and the *y*-axis represents average lagged EC throughout all duets in each condition. EC is essentially symmetric around 0 seconds (simultaneous playing) for *coupled* trials (red curve), but significantly higher in ghost-to-live (positive) lags compared to live-to-ghost (negative) lags for *one-way* trials (blue curve) (effect of lag sign on EC averaged across negative and positive lags: *M* = −2.90e-3, *SD* = 1.18e-3, 95% CI = [−5.22e-3, −5.35e-3]). This asymmetry in *one-way* trials was also found with respect to Combined Consonance (effect of lag sign on average CC: *M* = −3.45e-3, *SD* = 1.45e-3, 95% CI = [−6.32e-3, −6.19e-4]). These results reflect the underlying causal entanglements of each condition. Live musicians in *one-way* trials responded to ghost recordings by harmonizing with their past notes, but ghost recordings could not respond to notes of live musicians. There was no such asymmetry in *coupled* trials, because musicians were mutually responsive. As suggested by the difference in height between red (coupled) and blue (one-way) data points in Figure 3, EC was significantly higher overall in *coupled* versus *one-way* duos (effect of condition on simultaneous EC: *M* = −1.09e-2, *SD* = 4.91e-3, 95% CI = [−2.05e-2, −1.01e-3]), although this effect was not significant with respect to Combined Consonance (*M* = −1.51e-2, *SD* = 9.50e-3, 95% CI = [−43.41e-2, 3.21e-3]). In sum, coupled improvisers mutually harmonized with one another’s preceding notes, and this dynamic supported more consonant harmonization between them.

## DISCUSSION

This study examined how music produced by collaboratively improvising musicians is shaped by underlying patterns of coordination. Professional jazz pianists improvised in two duo conditions: a coupled condition in which they improvised together simultaneously, and an “overdubbed” (one-way) condition which precluded mutual adaptation because improvisers were recorded sequentially. Our analyses show that coupled duos achieved greater alignment of their note onsets and more consonant tonal coordination. These results were paralleled in the subjective experience of the performers and naive listeners, who preferred coupled duets despite being blind to condition.

Performers and listeners demonstrated systematic insight into the different causal entanglements of each condition. Leadership was rated as evenly distributed amongst coupled duos, but listeners perceived “ghost partners” as leading live musicians and performers rated themselves as playing more of a follower/supporter role in one-way duets. These listener results are remarkable in light of the fact that they were unable to guess which condition music samples were produced in above chance-level. Listeners were thus implicitly influenced by the presence or absence of mutual coupling, without their conscious awareness.

Coupled duos synchronized their note onsets more precisely than one-way duos, as in previous studies which showed that bidirectional coordination promotes synchronization in finger-tapping tasks (Konvalinka et al., 2010) and scored music performance (Demos et al., 2017; Goebl & Palmer, 2009). Here this phenomenon is observed in freely improvising musicians, with no explicit synchronization objective. Rather, precise synchronization emerged spontaneously, in service of the higher-level goal of collectively generating compelling music. Previous findings have also suggested that humans have an innate predisposition to entrain rhythms in social contexts (Kirschner & Tomasello, 2009), which could elucidate our result insofar as pianists may have sensed a lack of live responsiveness in their partners in one-way duets.

Mutual coupling supported note onset alignment at longer timescales as well. A cross-correlation analysis of onset density revealed that improvisers tended to match the rhythmic activity of their partners, and this tendency was significantly stronger in coupled duos. This relates to findings in non-musical JA domains. Previous dyadic conversation studies have shown that people spontaneously entrain their movements, and mimic one another’s facial expressions, manual gestures, eye gaze, and acoustic speech characteristics when verbally interacting with one another (Abney et al., 2014; Louwerse et al., 2012; D. C. Richardson & Dale, 2005; Shockley et al., 2003, 2009). Behavioral alignment has been proposed to foster successful interaction by signaling affiliative attitudes (Demos et al., 2012; Hove & Risen, 2009), and offloading predictive emulation (i.e., of a conversation partner’s future behavior) onto one’s own behavior (Garrod & Pickering, 2009); the temporal alignment observed here may serve these same interpersonal functions in improvised musical interactions.

Our onset density cross-correlation analysis also inferred different profiles of directional influence for each interaction condition. Cross-correlation was symmetric between coupled partners, but there was an asymmetry in one-way duos such that onset density of the live musician correlated with past onset density of the ghost partner (prerecorded track) but not vice versa. This result adds to previous demonstrations that causal influence in performing music ensembles is reflected in the movements and music of co-performers. This has been shown numerous times in the context of composed music (Chang et al., 2017; Demos et al., 2017; Keller & Appel, 2010), and the work of (Aucouturier & Canonne, 2017) suggested that leader-follower roles induced by experimentally manipulated social attitudes (e.g., caring, dominant) are reflected in sound envelopes (loudness) of improvising musicians. However, this latter finding was somewhat speculative because inter-musician coupling was not explicitly manipulated. In contrast, our overdubbed interaction condition provides a ground-truth to verify our analysis against.

Analogous findings were uncovered in the realm of abstract tonal structure. A novel lagged consonance analysis demonstrated that musicians harmonized with past notes of their partners. This occurred mutually in coupled duos but asymmetrically in one-way duos, where live musicians harmonized with preceding notes of the ghost recording, but not vice versa. Causal influence between improvisers was thus reflected not just in their rhythms, but also in the notes they played and the directed exchange of tonal information. Additionally, simultaneous EC was significantly greater in coupled duos, suggesting that the ability to mutually adapt to one another’s previous notes promoted robust tonal coordination.

Importantly, our consonance analysis detected not just alignment, but complementary tonal coordination as well. Consonance is not only achieved when musicians play the same pitch, but also when they play complementary sets of pitches that combine to produce consonant harmonies. The tonal coupling observed here can be understood in terms of interpersonal synergies, which have been proposed to emerge in interacting groups whose individuals co-constrain one another in support of group-level objectives (Fusaroli & Tylén, 2016; Hasson & Frith, 2016; Riley et al., 2011). In this case, note selection is co-constrained between collaboratively improvising musicians in order to generate tonal structure. Our consonance analysis contributes an important extension to previous analyses of naturalistic JA, which have primarily operationalized coordination in terms of behavioral matching, using techniques like cross-correlation and cross recurrence analysis (Dale & Spivey, 2006; Louwerse et al., 2012; Paxton & Dale, 2013; D. C. Richardson & Dale, 2005; D. C. Richardson et al., 2007). Here we demonstrate the feasability of using domain-specific measures (i.e., a tonal consonance model informed by music theory) to assess complementary coordination in support of abstract, functional properties at the group-level (i.e., emergent tonal structure). While there can be no doubt that alignment is an important interpersonal mechanism, more work of this kind is needed to investigate complementary coordination in naturalistic JA contexts (Hasson & Frith, 2016).

Successful coordination is difficult to operationalize in freely improvised music, because it is not explicitly clear what the intentions of musicians are. We analyzed rhythmic alignment and tonal consonance because they are basic musical elements, and we were able to operationalize them while imposing minimal musical assumptions (atonal music would be rated low consonance, onset density works for pulsed and nonpulsed music). The goal of participants was to generate compelling music, as they would strive for in a typical performance, but they were not explicitly instructed to synchronize note onsets or produce consonant harmonies. In fact, some level of musical tension and dissonance is typically desired. This being said, we observed robust effects that mutual coupling promoted temporal alignment and emergent tonal consonance overall. We also observed directional effects on these features consistent with the ground-truth unidirectional influence from recording to musician in one-way duets. Furthermore, these results were paralleled in the subjective experience of professional improvisers and naive listeners with no particular background in jazz music, who preferred mutually coupled duos, and correctly inferred leadership roles in both conditions.

Taken together, these results suggest that coupled dyads achieved enhanced, bidirectional temporal and tonal coordination, which supported the higher level goal of generating compelling music. This extends previous investigations of mutual coupling in idealized experimental paradigms, such as finger tapping (Konvalinka et al., 2010) and the improvised mirror game (Noy et al., 2011), into the rich, naturalistic setting of unconstrained musical improvisation. More specifically, our findings directly implicate the common studio recording technique of overdubbing—which we show results in systematically different music than live, coupled interaction. Lastly, our measures of expert musical coordination can be incorporated into the design of generative AI music systems to make them more humanlike and more musical (Datseris et al., 2019; Gillick et al., 2019; Hawthorne et al., 2019; Hennig, 2014; Hennig et al., 2011; Huang et al., 2018; Huang et al., 2019).

## ACKNOWLEDGMENTS

The authors thank Douglas Hofstadter for his generous support of this work. We also thank members of the Geolab and Evolutionary and Adaptive Systems (EASy) labs at Indiana University, as well as Todd Gureckis’s Computational Cognitive Science lab at New York University for invaluable feedback. And of course, we thank all of the tremendous musicians who participated in this study.

## AUTHOR CONTRIBUTIONS

MS: Conceptualization: Lead; Data curation: Lead; Formal analysis: Lead; Investigation: Lead; Methodology: Equal; Project administration: Lead; Resources: Lead; Software: Lead; Visualization: Lead; Writing - Original Draft: Lead; Writing - Review & Editing: Lead. RG: Conceptualization: Supporting; Formal analysis: Supporting; Funding acquisition: Lead; Methodology: Equal; Resources: Supporting; Supervision: Lead; Validation: Lead; Visualization: Supporting; Writing - Review & Editing: Supporting.

## Notes

^1^ Musical Instrument Digital Interface (MIDI) is a format for representing music on a computer. It symbolically represents the pitch, volume and timing (onset and offset) of musical note sequences.^2^ “Duo” refers to a pair of performers and “duet” refers to a particular piece produced by a duo. Each *coupled* duo yoked two *one-way* duos, same for duets.^3^ A range of window sizes (2, 5, and 10 s) were evaluated, with a hop size of 2 s. The following reported results were robust across all window sizes.
