# Supplementary material for: Coordination and Consonance Between Interacting, Improvising Musicians
Source: Open Mind (Camb). 2020 Nov 1;4:88–101. doi: 10.1162/opmi_a_00036 (PMC8412203; doi:10.1162/opmi_a_00036)
Supplement: Supplementary file 1 [file opmi-04-88-s001.pdf]

Supporting Information: Coordination and Consonance Between Interacting, Improvising Musicians

Matthew Setzler & Robert Goldstone

Data and Analysis Scripts

The complete MIDI dataset, as well as preprocssing and analysis scripts can be accessed here: https://osf.io/wxf4s/.

Methods

Listener Study. A post-hoc study was conducted with populations of naive listeners and expert jazz musicians. Listeners heard 30-second audio clips randomly sampled from duets in both conditions. After listening to each clip they were asked to rate (1) their enjoyment of the music (2) how well coordinated they perceived the musicians to be and (3) which musician played more of a leader role. Listeners were also asked to guess which condition a clip came from. Each listener heard a sequence of 24 audio clips, 12 from each condition. Audio clips were randomly sampled so that every participant heard a different set of clips. Clips were sequenced in two complementary blocks. Block 1 contained 12 random clips (6 from each condition) and the Block 2 contained complements, or correspondingly yoked clips of Block 1 (i.e. *one-way* clips yoked from coupled clips in Block1, and vise versa). This design controlled for variability between trials, and allowed us to compare within-subject ratings of yoked music clips from both conditions. Clips were randomly ordered within each block to control for order effects. Individual music streams were panned to separate ears, and counterbalanced across *one-way* clips so that ghost recordings were panned to each ear 50% of the time.

Apparatus. Two electric keyboards were used: a Roland Juno-Di and Nord Electro 2, both of which had 61 semi-weighted keys. Both keyboards were used on every trial (i.e. *one-way* trials were arranged such that live pianists played whatever keyboard their ghost partner did not play). Ableton Live 9 Lite (running on a MacBook Air) was used to collect isolated MIDI recordings, and synthesize the audio that participants heard in the style of an electric Rhodes. This ensured time alignment of MIDI recordings, and that participants heard the same exact timbre for themselves and their partner, irrespective of condition. Participants were recorded at a music studio in Brooklyn, NY. The studio was divided by a curtain preventing participants from seeing one another, and participants heard themselves and their partners through Sony CH700N Noise Cancelling headphones. Participants thus had no visual or audible indication of their condition on a given trial.

Tonal Consonance Model. Our measure of tonal consonance was adapted from a previous model of musical tonality called the Tonal Spiral Array, which has been validated against listener ratings and expert music theory analyses of musical tension (1, 2). While there are alternative models of tonal consonance to consider (3, 4), the Tonal Spiral Array is especially appropriate in the context of freely improvised music because has been validated with respect to both functional and post-tonal music (5), and has the flexibility to model temporally extended (as opposed to just instantaneous) tonal structure (1). We adapted the "cloud diameter" measure of tonal dissonance (2) to create conditions of enharmonic equivalence (e.g. D# and Eb are treated equivalently in our measure).

The rationale behind the consonance measure is that certain pairwise pitch intervals are inherently more or less consonant. For example, a tritone (e.g. {C,F#}) is highly dissonant (lowly consonant), whereas a perfect 5th (e.g. {C,G}) is highly consonant (lowly dissonant). Accordingly, every interval class was assigned a 'dissonance rating', as indicated in Figure 3A (2). These dissonance ratings were taken from previous applications of the Tonal Spiral Array (2, 5), as the closest euclidean distance between pitch pairs for each interval. Tonal consonance was computed for a given window of music as the negative weighted sum of dissonance ratings, scaled by the normalized frequency at which intervals occurred within said window (i.e. such frequencies of all interval classes summed to 1). In scaling by normalized frequency we ensured that there was no correlation between consonance and the amount of sustained sound within a window. Details of how normalized frequency distributions were obtained are depicted in Figure 4. Lastly, a constant of 2 was added to avoid negative values, since consonance is the opposite of dissonance. Table 1 shows model ratings for exemplar pitch sets.

Table 1. Consonance ratings of exemplar pitch sets.

Pitch Set	Consonance
{C,E,G} (Cmaj)	.65
{C,Eb,G} (Cmin)	.65
{C,B,G}	.54
{C,E,G,F,A,C} (Cmaj + Fmaj)	.49
{C,B}	.48
{C,E,G,F#,A#,C#} (Cmaj + F#maj)	.13
serial (all 12 pitches)	.09

Consonance time series were computed from music streams using 2, 5 and 10 second sliding windows with 2 second hop size, as illustrated in Figure 3B. Three measures of consonance were considered: Individual Consonance (consonance of individual music streams), Combined Consonance (consonance of merged music streams from both players in a dyad) and Emergent Consonance (Combined Consonance minus average Individual Consonance of both musicians in a dyad). *Emergent consonance* is essentially a measure of tonal coordination, as it captures the consonance arising from the *interaction* of pitches played by the two different musicians. A situation in which each pianist plays consonant notes that clash with one another would result in low emergent consonance (e.g. {C,E,G} and {F#,A#,C#} are consonant on there own but {C,E,G,F#,A#,C#} is highly dissonant), whereas a situation in which each pianist plays dissonant pitch sets that stabilize one another when sounded together would result in high emergent consonance (e.g. {C,B} and {E,G} have low average consonance but {C,E,G,B} has high consonance because it is tonicized to a Cmaj7 chord).

Results

Performer Subjective Responses. At the conclusion of every trial participants filled out a questionnaire indicating their subjective experience of (1) quality of the improvised piece (2) how well coordinated they were with their partners (3) how easy it was to coordinate with their partners and (4) the degree to which they felt they played a leader or a supporter role. Responses to questions (1-3) were given on a 5 point Likert scale and responses to question 4 were given on a 5 point scale in which 1 corresponded to a strong leader role and 5 corresponded to a strong supporter role. Responses are depicted in Figure 5.

Tempogram Analysis. A tempogram analysis was conducted to determine the extent to which duets were rhythmically pulsed with a steady beat (i.e. "in time" as opposed to "out of time"). The librosa library in python was used to obtain tempograms from audio recordings of duets in both conditions (6, 7). Tempograms were then summed to give an overall score of "pulsedness" per duet. To test for effects of interaction condition, a mixed-effects model was fit to predict pulsedness as a function of condition, with random intercepts for yoked groupings at the duo and duet levels. This analysis did not indicate a significant effect of condition (estimate of condition slope: M = -425.07, SE = 13412.70, $t(86.88)=-0.023$, $p>0.1$), thus we conclude that *coupled* and *one-way* duets were equally pulsed. This model was fit using the lme4 package in R, and p value was obtained using lmerTest, which uses Satterthwaite's method to estimate degrees of freedom (8, 9).

Cross-correlation of co-performer onset density. Cross-correlation of onset density decreases for large-magnitude lags, as depicted in Figure 6.

Relationship between onset density and tonal consonance. Although our measure of tonal consonance normalized for the combined duration of sustained notes, it did not explicitly normalize for onset density. Accordingly, we examined the correlation between consonance and onset densit in individual music streams. The relationship between these measures is depicted in Figure 7. Overall these plots indicate a negative correlation between consonance and onset density (correlation = .377, 95% CI=[.37,.38], $t(299466)=222.73$, $p < 0.01$), although there is considerable spread of consonance especially at low levels of onset density which are more common in our dataset. Such a negative correlation is unsuprising and a desired property: in time windows with more notes played (i.e. higher onset density) there are more opportunities for dissonant intervals to occur, resulting in more dissonance probabilistically. Nonetheless, this correlation made it necessary to deconfound effects of tonal consonance from those of onset density, as reported in subsequent supplementary analyses.

Granger Causality Between Co-Performers' Onset Density and Tonal Consonance. The Multivariate Granger Causality (MVGC) toolbox in MATLAB was used to compute Granger Causality (GC) amongst co-performers' onset density and tonal consonance time series (10). This toolbox allowed us to deconfound the correlation between onset density and tonal consonance by separately computing pairwise GC between onset density (conditioned on tonal consonance) and consonance (conditioned on onset density).

Data pre-processing and granger causality computation. Onset density and Consonance time series were obtained from each individual MIDI time series with 2 second windows sliding windows and 0.6 second step sizes. These time series were detrended with first differencing to remove non-stationarity. We had to account for missing values in Tonal Consonance time series, because

Consonance is undefined in time windows with no playing. Accordingly, we subtracting the temporal mean of each consonance time series from every value, and set missing values to 0.

Once the data was pre-processed, pairwise GC between co-players' Onset Density and Consonance time series was computed separately for each trial, following the procedures of the MVGC toolbox demo. This included a test to ensure time series passed the Granger causality stationarity assumption, with spectral radius of less than one. This condition was met by time series in all trials except for 2, which were discarded from further analysis.

GC computation involves a model comparison between a restricted Vector Autoregressive Model (VAR) (e.g. predict A from A's past) with a full VAR model (e.g. predict A from A's past *and* B's past). As suggested in the literature (10), these should each have the same model order. GC was computed repeatedly for each trial over a range of hand-chosen model orders. Since our time series came from sliding windows, VAR models with small orders would be fit with data in which there is substantial temporal overlap between predictors and predicted values. We avoided this issue by hand-choosing reasonable model orders that were sufficiently high to avoid such temporal overlap. Moreover, in computing GC for each trial over a range of model orders we were able to see how robust GC results were to model order.

Results and Analysis. GC was compared across three conditions: ghost-to-live (GC from ghost recording to live musicians in one-way trials), live-to-ghost (vise versa) and live-to-live (granger causality between mutually interacting musicians in coupled trials). The main GC output was a log-likelihood model comparison of full and restricted models, which we used as a continuous dependent measure of causality as has been done in previous applications (11). Results for are depicted in Figure 8.

Overall there is higher GC of onset density compared to tonal consonance. GC of onset density reflects the underlying patterns of influence enforced by each condition. Within *one-way* trials, GC of ghost-to-live was higher than live-to-ghost (paired $t(84)=3.724$, $p < 0.01$; comparing gc values in each direction within each one-way trial, and using GC values obtained with 5.4 second model order). Thus, onset density of live musicians in *one-way* trials was responsive to that of the ghost recordings they were playing with but not the other way around. There was no significant difference in GC between ghost-to-live and live-to-live (i.e. coupled) conditions ($t(283)=.101$; $p=.92$; 5.4 second model order).

Granger causality has been applied in related work to show that leader follower relations in music ensembles playing composed music are reflected in the postural sway of performers (11, 12). Here we find a related to collectively improvised musical structure: experimentally manipulated conditions of interaction constrain the directed flow of musical information (such as onset density) within dyads of improvising pianists, and these different patterns of causal influence can be detected with Granger causality.

Lagged Consonance. In the main body of this paper we report that within *one-way* trials lagged Emergent Consonance are both higher at ghost-to-live lags versus live-to-ghost lags. This result was robust over the full range of window sizes, and also held for lagged Combined Consonance, as depicted in Figure 11. Table 2 reports posterior estimates for Bayesian mixed-effects models predicting either Emergent or Combined Consonance in one-way duets as a function of lag sign. Separate models were fit for each window size. Table 3 reports posterior estimates for Bayesian mixed-effects models predicting simultaneous Emergent Consonance (EC) and Combined Consonance (CC) as a function of condition. EC is significantly higher in coupled duets across all window sizes, but this effect is not significant across all window sizes for Combined Consonance.

Table 2. Lagged consonance for one-way duets. (*) denotes >95% confidence that predictors are below zero, (.) denotes >90% confidence that fixed-effect predictors are below zero. (Significance of Intercepts is not marked.)

Predictor	M	SD	95% CI-lower	95% CI-upper
Combined consonance - 2 second window				
Intercept	0.2873	0.0104	0.2673	0.3078
Direction *(ghost-to-live versus live-to-ghost)	-0.0036660846	0.0014576061	-0.0065091675	-0.0008027151
Combined consonance - 5 second window				
Intercept	0.2515	0.0111	0.2298	0.2728
Direction *	-0.0034494142	0.0014512443	-0.0063187044	-0.0006185724
Combined consonance - 10 second window				
Intercept	0.2273	0.0108	0.2063	0.2491
Direction *	-0.0031450109	0.0013200759	-0.0056900219	-0.0005003359
Emergent consonance - 2 second window				
Intercept	-0.1334377293	0.0038863905	-0.1410237049	-0.1257961209
Direction *	-0.0028700801	0.0011999974	-0.0052312859	-0.0005354221
Emergent consonance - 5 second window				
Intercept	-0.1046593839	0.0042015904	-0.1132617365	-0.0967792528
Direction *	-0.0028987367	0.0011839063	-0.0052187113	-0.0005353739
Emergent consonance - 10 second window				
Intercept	-0.0867296305	0.0045040980	-0.0957039390	-0.0782383728
Direction *	-0.0027763878	0.0010978847	-0.0049083277	-0.0005766808

Given the correlation between tonal consonance and onset density, we performed a supplementary analysis to confirm that these results are not somehow artifactual of condition-wise differences in onset density. The same lagged analysis was performed

Table 3. Simultaneous consonance by condition. (*) denotes >95% confidence that fixed-effect predictors are below zero, (.) denotes >90% confidence that predictors are below zero.

Predictor	M	SD	95% CI-lower	95% CI-upper	Pr(<0)
Combined consonance - 2 second window					
Intercept	0.3098	0.0145	0.2812	0.3394	
Condition . (coupled versus one-way)	-0.01332	0.00895	-0.03066	0.00526	.93
Combined consonance - 5 second window					
Intercept	0.2765	0.0152	0.2457	0.3059	
Condition .	-0.01509	0.00950	-0.03405	0.00321	.946
Combined consonance - 10 second window					
Intercept	0.2533	0.0147	0.224	0.2824	
Condition *	-0.01679	0.00932	-0.03479	0.00194	.963
Emergent consonance - 2 second window					
Intercept *	-0.130314	0.006551	-0.143290	-0.116953	
Condition *	-0.010848	0.004922	-0.020359	-0.001295	.99
Emergent consonance - 5 second window					
Intercept	-0.10235	0.00825	-0.11802	-0.08635	
Condition *	-0.01087	0.00491	-0.02046	-0.00101	.99
Emergent consonance - 10 second window					
Intercept	-0.085054	0.008705	-0.102079	-0.068029	
Condition *	-0.010449	0.005134	-0.020597	-0.000465	.98

with respect to combined onset density instead of combined/emergent consonance. If onset density was driving the asymmetry in *one-way* trials we would expect to see a similar asymmetry in *one-way* with onset density (though in the opposite direction, because of the negative correlation). There was no such asymmetry (paired t(85)=1.197, p=0.235), indicating that the above results are genuinely reflective of tonal coordination.

Disentangling Alignment and Complementary Tonal Coordination. It was demonstrated in the main body of the text that mutual coupling promotes tonal coordination, in that *coupled* duos exhibited greater Emergent Consonance compared to *one-way* duos. But is this effect due to greater tonal alignment, in the sense that coupled musicians are more likely to play more of the same pitches as one another, or is it due to *complementary* tonal coordination, in the sense that musicians play different notes that consonantly harmonize together? To disentangle pitch matching from complementary tonal coordination, we analyzed Tonal Entropy of the combined notes produced by duos as a function of interaction condition. Low tonal entropy of the combined notes of a duo indicates that musicians were aligning on a restricted subset of the 12 pitch classes (indicative of pitch matching) whereas high tonal entropy indicates that pitch content was more evenly distributed across the 12 pitch classes. Tonal Entropy was computed over the distribution of all 12 pitch classes in the chromatic scale using Shannon's information theory definition of entropy (13). For a given time window, a probability distribution was obtained by incrementing bins of each pitch class by the number of note onsets musicians played from that pitch class*. This was done repeatedly with 2, 5 and 10 second sliding windows and a hop size of 200 milliseconds, yielding time series of Tonal Entropy for each duet. A Python implementation of this measure can be found in the osf directory linked above.

Bayesian mixed-effects models were fit to predict Tonal Entropy (average Tonal Entropy throughout each duet) as a function of interaction condition, with random effects for yoked groupings at the pair and trial levels. Results are depicted in Table 4. While not entirely robust over all window sizes, these posterior estimates show that Tonal Entropy is generally lower in *coupled* duets (though this effect was not significant in 2 second window sizes), thus suggesting that mutual coupling promotes tonal alignment.

Table 4. Posterior estimates of Bayesian multi-level modeling predicting Tonal Entropy from interaction condition. (*) and (.) denote >95% and >90% confidence, respectively, that Tonal Entropy is lower in *coupled* trials.

Predictor	Window Size (sec)	M	SD	95% CI-lower	95% CI-upper
Intercept	2	3.1789	0.0193	3.1409	3.2162
Condition (coupled versus one-way) .	2	0.0225	0.0141	-0.0049	0.0505
Intercept	5	3.1325	0.0264	3.0793	3.1837
Condition *	5	0.0351	0.0191	-0.0026	0.0726
Intercept	10	3.1319	0.0313	3.0703	3.1935
Condition *	10	0.0469	0.0215	0.0044	0.0900

This being said, we were still interested in whether mutual coupling also promotes greater *complementary* tonal coordination, especially given that the effect of interaction condition on Emergent Consonance was more robust than it was on Tonal

* A prior uniform distribution was assumed by initializing probability bins for each pitch class to 0.5. This was done to ensure that passages with just one or two instances of a particular note onset weren't assigned disproportionately low entropy.

Entropy. Two Bayesian multi-level models were fit and analyzed to disentangle the relative contributions of matching versus complementary tonal coordination with respect to the observed Emergent Consonance results. A restricted model predicted Emergent Consonance as a function of Tonal Entropy (with random effects at the pair and trial level) and a full model predicted Emergent Consonance as a function of both Tonal Entropy and interaction condition (with the same random effects). To the degree that the full model outperforms restricted model, we can infer that mutual coupling promotes complementary tonal coordination resulting in higher EC. This is because Tonal Entropy is a measure of pitch matching, and if EC was fully explained by matching alone, condition effects would already be encapsulated in the entropy predictor.

Posterior estimates, displayed in Table 10b, reveal that Tonal Entropy did not significantly predict EC in the restricted or full models for 2 and 5 second windows, (although it did for 10 second windows), whereas interaction condition significantly predicted EC in full models across all window sizes. The loo_compare method implemented in brms R package was used to perform model comparison between restricted and full models, using the Watanabe–Akaike information criterion (WAIC) (REF?). This model comparison revealed that full models outperformed restricted models at each time window, as summarized in Table 6. This analysis thus reveals that the previously observed effect of interaction condition on Emergent Consonance is not merely a consequence of greater tonal alignment between mutually coupled musicians, but also from greater complementary tonal coordination between mutual coupled improvisers, who play different pitches that interact to produce more consonant harmonies.

Table 5. Posterior estimates for full and restricted models relating Emergent Consonance to Tonal Entropy. (*) mark fixed-effects predictors whose 95% Confidence Intervals are non-inclusive of zero.

Predictor	Window Size (sec)	M	SD	95% CI-lower	95% CI-upper
Restricted Model Fits					
Intercept	2	-0.18732	0.08990	-0.36809	-0.01121
Entropy	2	0.01591	0.02816	-0.03956	0.07271
Intercept	5	-0.19121	0.06755	-0.32389	-0.05975
Entropy	5	0.02624	0.02133	-0.01551	0.06774
Intercept	10	-0.19461	0.06053	-0.31210	-0.07418
Entropy *	10	0.03273	0.01899	-0.00509	0.06973
Full Model Fits					
Intercept	2	-0.20251	0.08751	-0.37385	-0.03235
Entropy	2	0.02288	0.02749	-0.03079	0.07724
Condition (coupled versus one-way) *	2	-0.01166	0.00490	-0.02126	-0.00199
Intercept	5	-0.20276	0.06606	-0.33272	-0.07571
Entropy	5	0.03230	0.02094	-0.00742	0.07337
Condition *	5	-0.01250	0.00502	-0.02236	-0.00260
Intercept	10	-0.20587	0.05862	-0.32084	-0.09021
Entropy *	10	0.03868	0.01852	0.00247	0.07514
Condition *	10	-0.01258	0.00521	-0.02278	-0.00221

Table 6. Model comparison between restricted and full models of Emergent Consonance. Expected Log Predictive Density (ELPD) used for model evaluation. Full model outperforms restricted model for each window size. Values indicate difference between metric for full model minus that of restricted model.

Window Size (seconds)	ELPD difference (full minus restricted model)	Standard Error of ELPD difference (full minus restricted model)
2	-2.5	2.7
5	-2.6	2.7
10	-2.5	2.5

Tests for Artifacts of Onset Density. Two supplementary analyses were performed to verify that these results were not artifactual of onset density. First we looked for correlation between onset density (combined within a dyad) and Emergent Consonance. As is displayed in Figure 9, there is no strong correlation between these features, except for extremely high values of onset density which were outliers. Second, we contrasted overall level of individual consonance between musicians playing in *coupled* and *one-way* trials. If EC results were artifactual of there being more onset density in *coupled* trials, we would expect lower individual consonance in *coupled* trials which would in turn result in higher EC. But, as depicted in Figure 10 this is not the case.

1. E Chew, , et al., Mathematical and computational modeling of tonality. *AMC* **10**, 12 (2014).
2. D Herremans, E Chew, , et al., Tension ribbons: Quantifying and visualising tonal tension. (2016).
3. P Harrison, M Pearce, Instantaneous consonance in the perception and composition of western music. (2019).
4. PN Johnson-Laird, OE Kang, YC Leong, On musical dissonance. *Music. Perception: An Interdiscip. J.* **30**, 19–35 (2012).
5. E Chew, Regards on two regards by messiaen: post-tonal music segmentation using pitch context distances in the spiral array. *J. New Music. Res.* **34**, 341–354 (2005).
6. B McFee, et al., librosa: Audio and music signal analysis in python in *Proceedings of the 14th python in science conference.* Vol. 8, pp. 18–25 (2015).
7. P Grosche, M Müller, F Kurth, Cyclic tempogram—a mid-level tempo representation for musicsignals in *2010 IEEE International Conference on Acoustics, Speech and Signal Processing.* (IEEE), pp. 5522–5525 (2010).

8. D Bates, M Mächler, B Bolker, S Walker, Fitting linear mixed-effects models using lme4. *arXiv preprint arXiv:1406.5823* (2014).
9. A Kuznetsova, PB Brockhoff, RH Christensen, , et al., lmertest package: tests in linear mixed effects models. *J. statistical software* **82**, 1–26 (2017).
10. L Barnett, AK Seth, The mvgc multivariate granger causality toolbox: a new approach to granger-causal inference. *J. neuroscience methods* **223**, 50–68 (2014).
11. A Chang, SR Livingstone, DJ Bosnyak, LJ Trainor, Body sway reflects leadership in joint music performance. *Proc. Natl. Acad. Sci.* **114**, E4134–E4141 (2017).
12. JJ Aucouturier, C Canonne, Musical friends and foes: The social cognition of affiliation and control in improvised interactions. *Cognition* **161**, 94–108 (2017).
13. CE Shannon, A mathematical theory of communication. *The Bell system technical journal* **27**, 379–423 (1948).

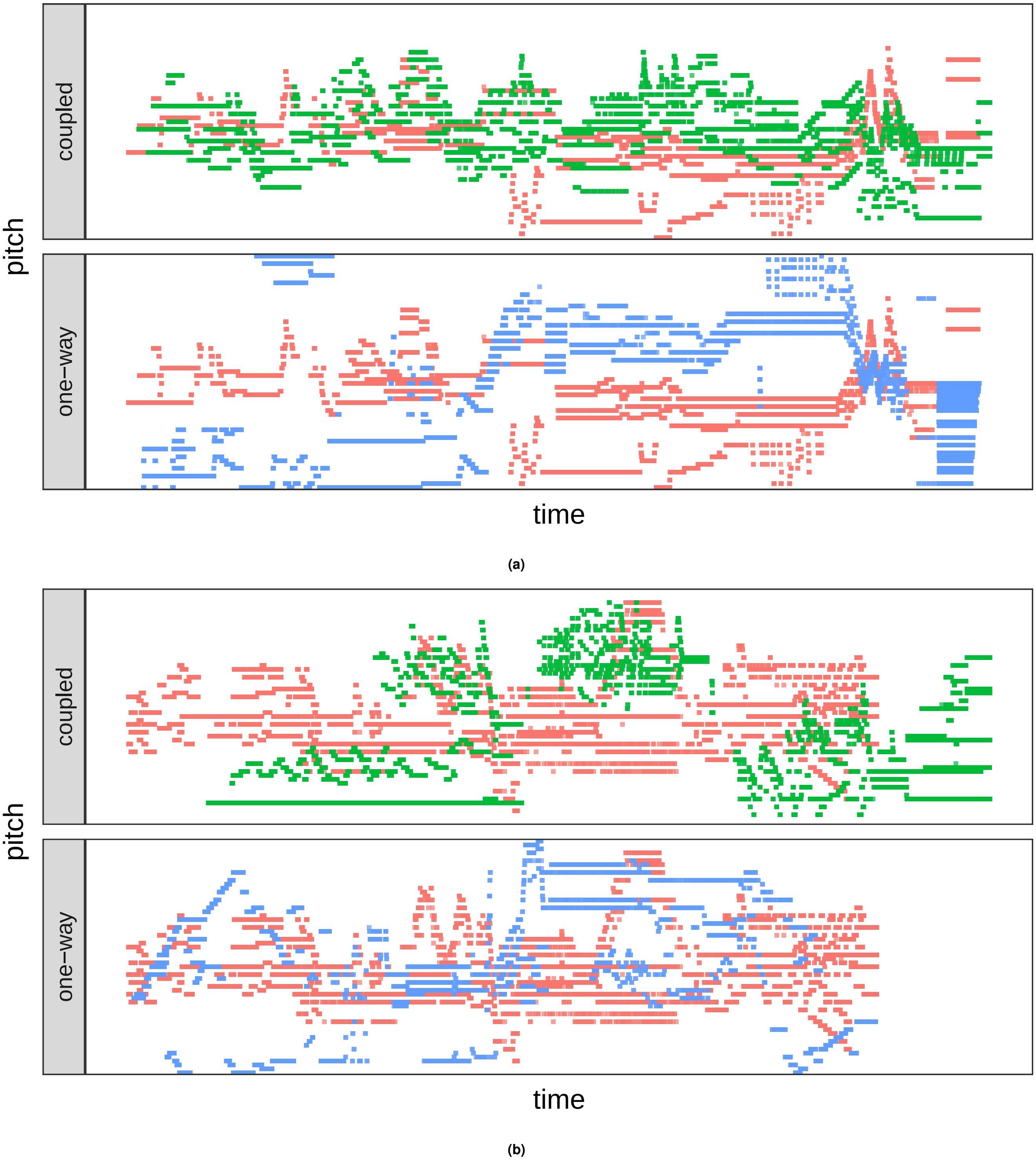

Fig. 1. Piano roll visualizations of exemplar yoked duets. x-axis represents time and y-axis represents pitch. Notes from separate musicians are colored differently. Each pair of piano rolls represent yoked duets; red musician from the top *coupled* duet was ghost partner in the bottom *one-way* duet.

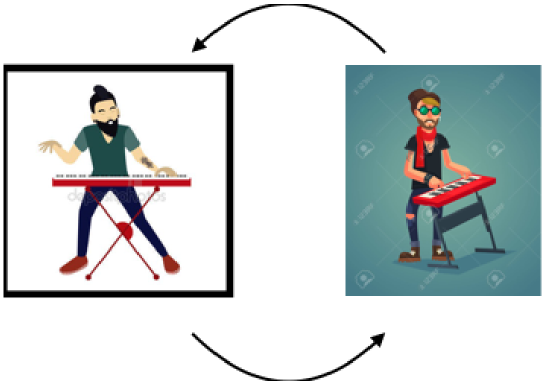

(a) *coupled* condition

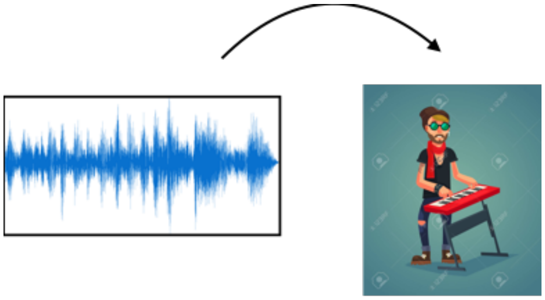

(b) *one-way* condition

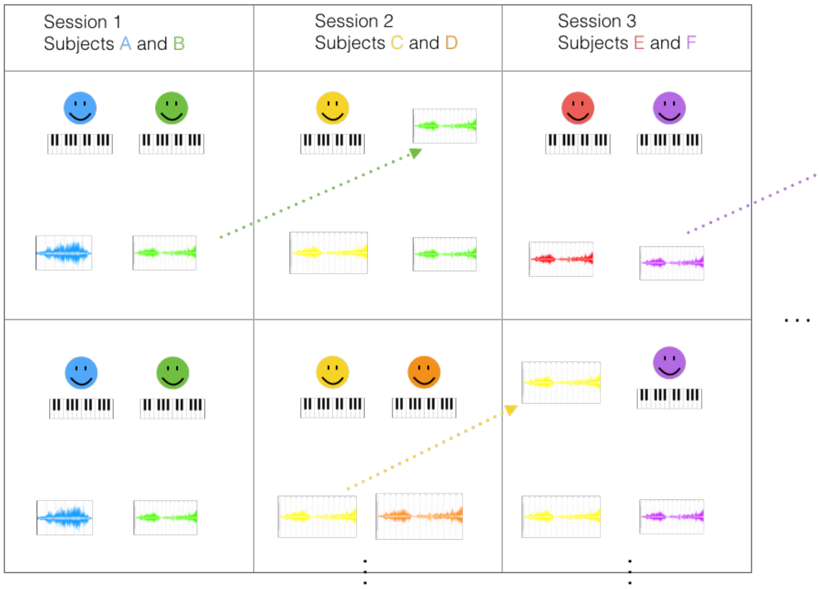

(c) Yoked experimental design. Pairs of participants entered the studio in separate sessions. Each participant played with the same live partner for all *coupled* trials and the same ghost partner for all *one-way* trials. Participants played at least 3 trials in each condition. Conditions were interleaved within sessions and counterbalanced across sessions to control for possible order effects. Individual tracks from each *coupled* trial were used to yoke *one-way* trials in the following session.

Fig. 2. Experimental procedure.

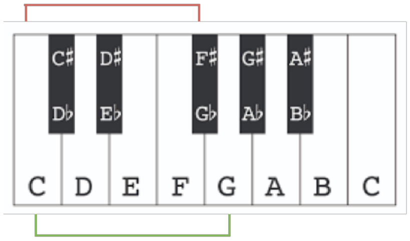

interval	dissonance
unison	1.0
m2/M7	2.6
M2/m6	2.2
m3/M6	1.9
M3/m6	1.7
P4/P5	1.5
A4	3.2

(a)

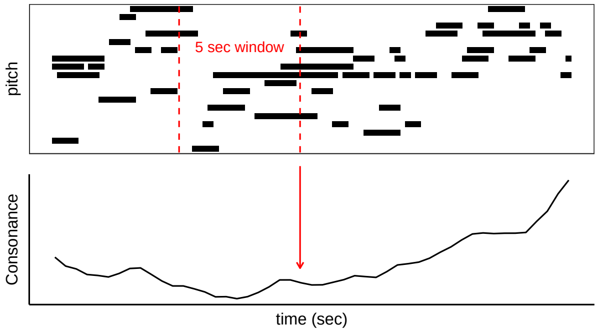

(b)

Fig. 3. Tonal consonance measure. (a) Every pitch interval is assigned a dissonance rating (perfect fifth and tritone are colored for illustration). Tonal consonance is the negative weighted sum of dissonance levels scaled by how often intervals occur within windows of music. (b) Consonance time series were computed from music sequences using 5 second sliding windows.

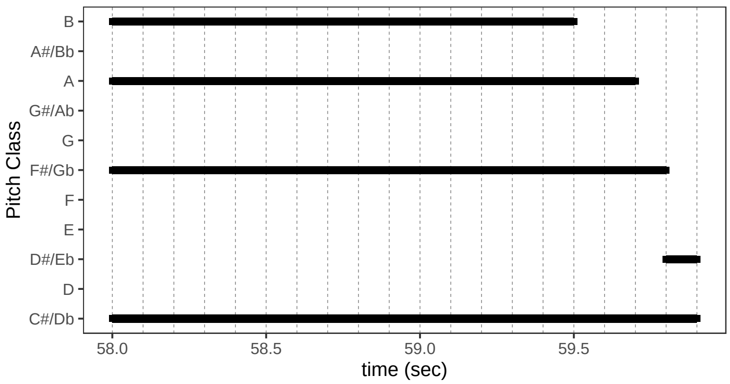

(a)

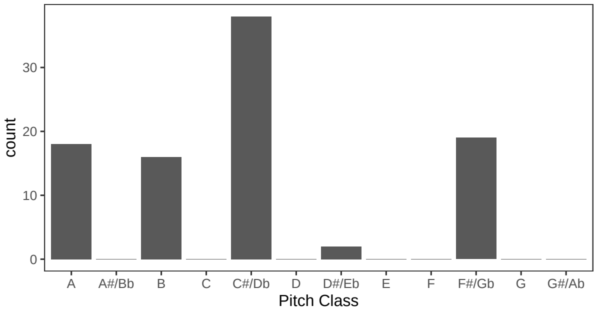

(b)

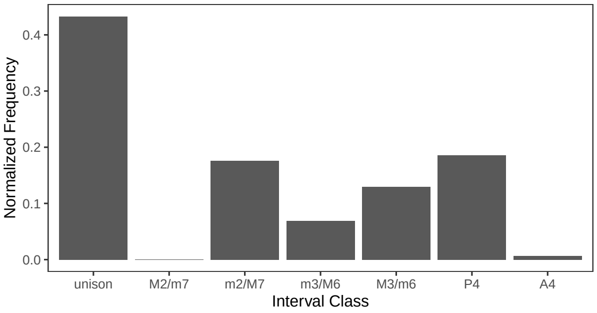

(c)

Fig. 4. Tonal consonance measure for arbitrary 2 second window of music. Frequency distributions for every pitch class (b) were obtained by splitting music windows into 200 millisecond bins and counting the number of bins where each piece class was active (a). Pitch class distributions were then used to determined normalized frequency distributions of intervals (c), by determining how much each interval class was active and normalizing such that frequencies of all intervals summed to 1.

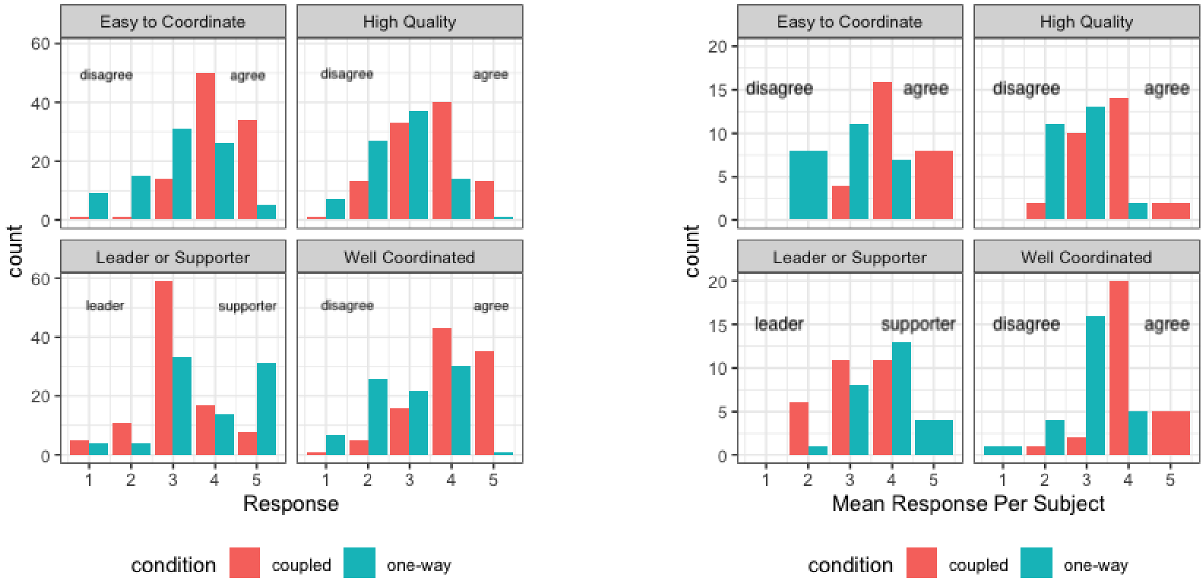

(a) **(b)**

Fig. 5. Subjective responses. (A) depicts counts of all participant responses across each condition and (B) depicts counts of the average response of every participant within each condition.

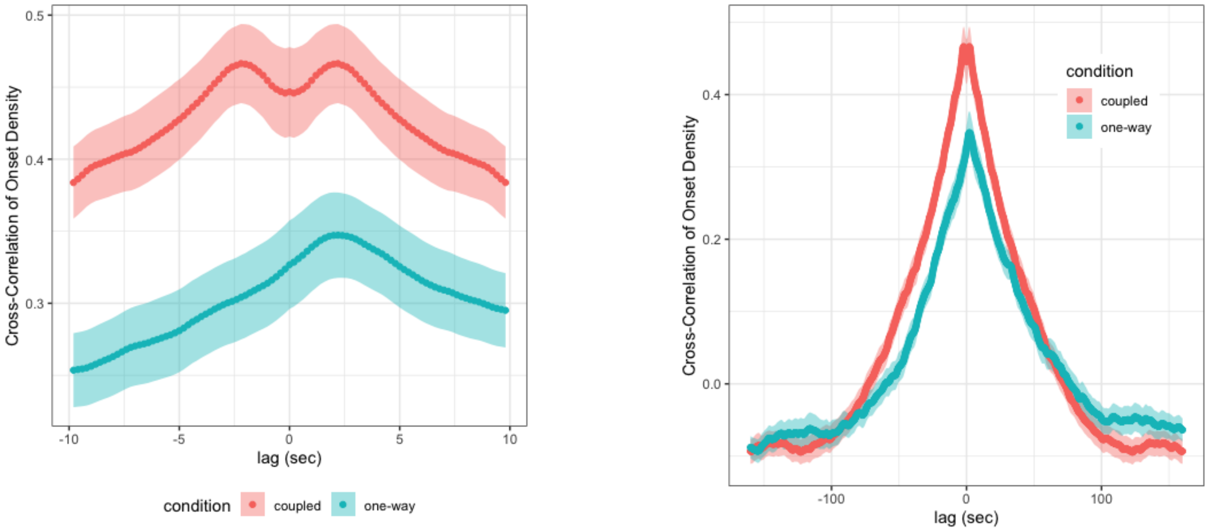

(a) **(b)**

Fig. 6. Cross-correlation (cc) of onset density between co-performers. Onset density was computed for individual music streams with a 2 second sliding window and 0.2 second hop size. (a) CC is higher overall in *coupled* trials. Also, within *one-way* trials cc is higher at positive lags (ghost-to-live) as opposed to negative lags (live-to-ghost), indicating that onset density of live performers responds to that of ghost recordings but not the other way around. (b) CC in two conditions converge at lags of large magnitude.

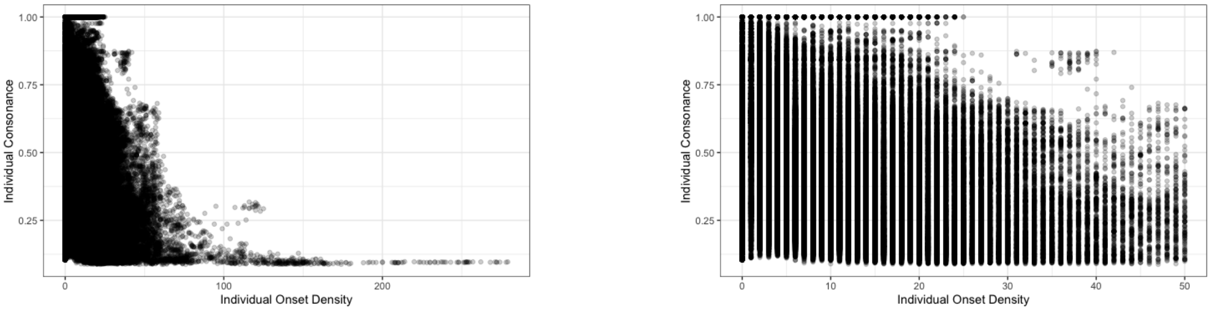

(a) (b)

Fig. 7. Relationship between tonal consonance and onset density in individual music streams. Each point represents onset density and tonal consonance values at simultaneous time indices within individual music streams.

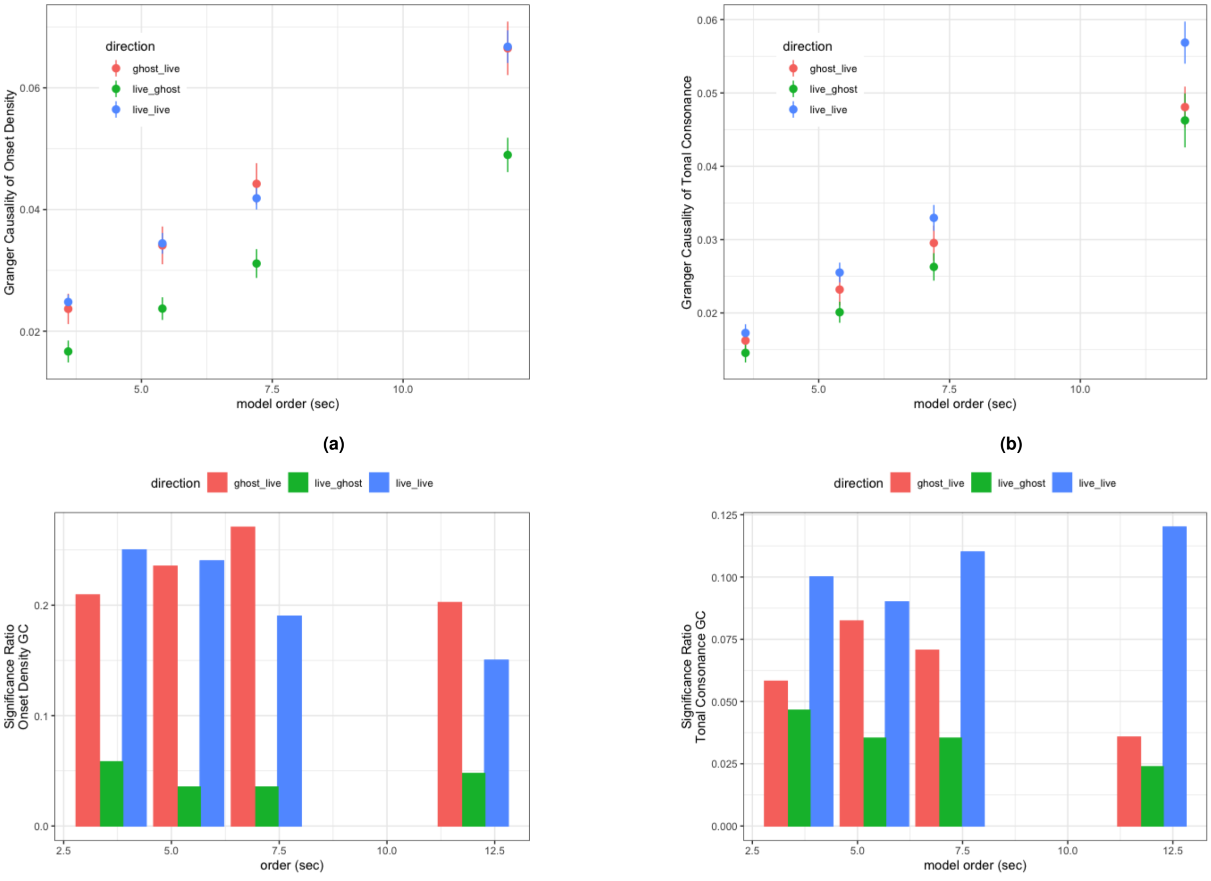

(c) (d)

Fig. 8. Granger causality reflects directed flow of improvised musical information. (A) and (B) depict mean GC of co-performer onset density and tonal consonance, respectively, for all trials within each condition computed across a range of model orders (x-axis). Error bars denote standard error. (C) and (D) depict the proportion of trials in which GC was statistically significant at 0.05 significance levels.

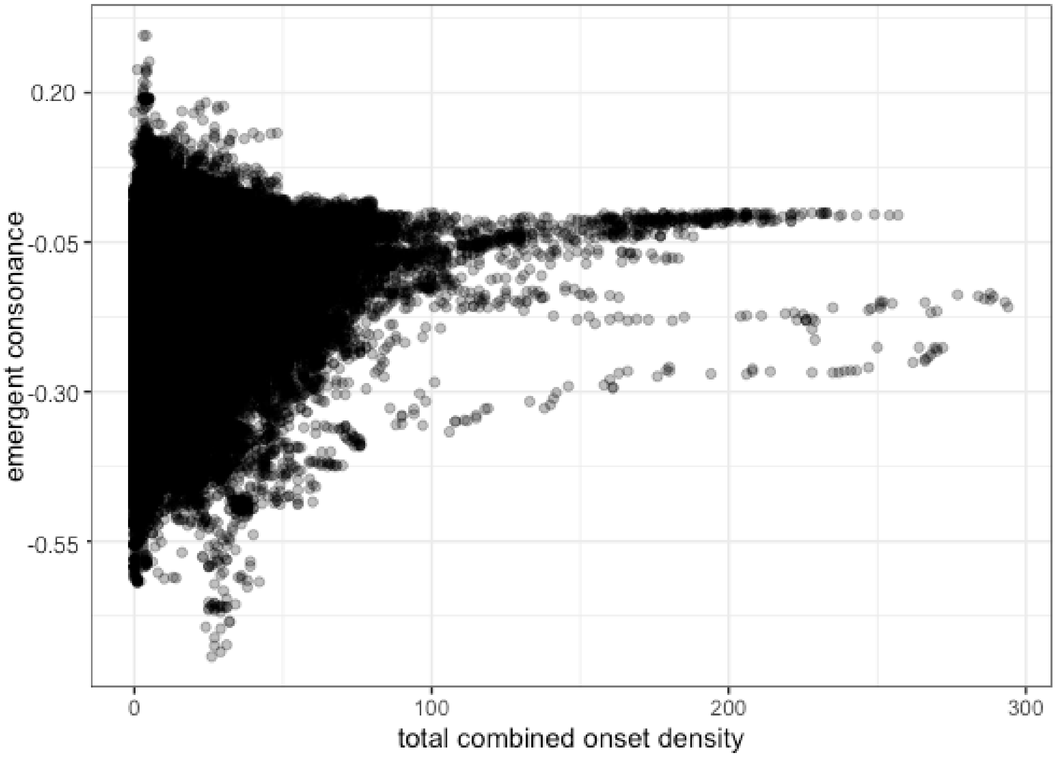

Fig. 9. Combined onset density and Emergent Consonance (EC). Each point depicts combined onset density (2 second sliding window with 0.2 second hop size) and EC at simultaneous time indices across all pieces. For the most part there is no correlation between onset density and emergent consonance, except for outliers of extremely high onset density.

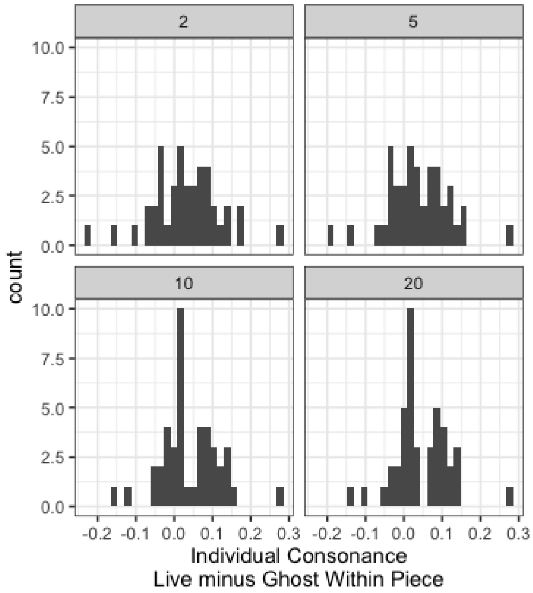

(a)

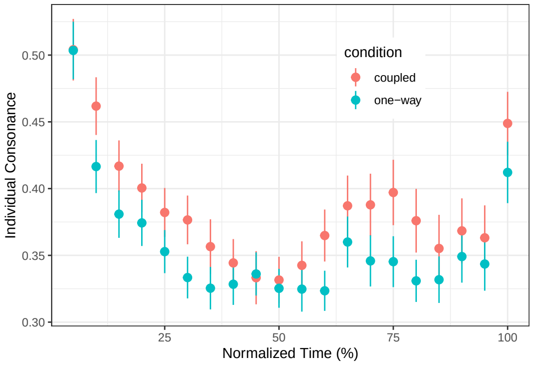

(b)

Fig. 10. Individual tonal consonance over time in *coupled* and *one-way* trials. (A) depicts the difference between average individual tonal consonance throughout *coupled* pieces minus that of the correspondingly yoked *one-way* pieces. This resulted in one value (*coupled* - *one-way*) for each yoked ID for every window size of tonal consonance (2, 5, 10 and 20 seconds all with 0.2 second hop size). Distributions are depicted for each window size. (B) Depicts individual tonal consonance (5 second sliding window) over normalized time. Time was normalized by dividing each piece into 20 equispaced temporal bins and averaging individual consonance within each bin. Points display mean consonance across all pieces within each condition and error bars denote standard error of the mean.

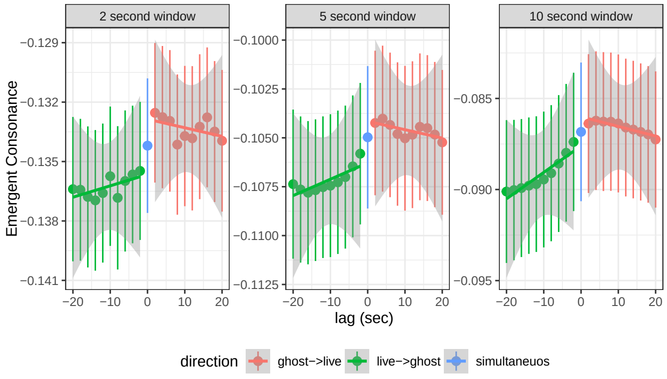

(a) Lagged emergent consonance in one-way trials.

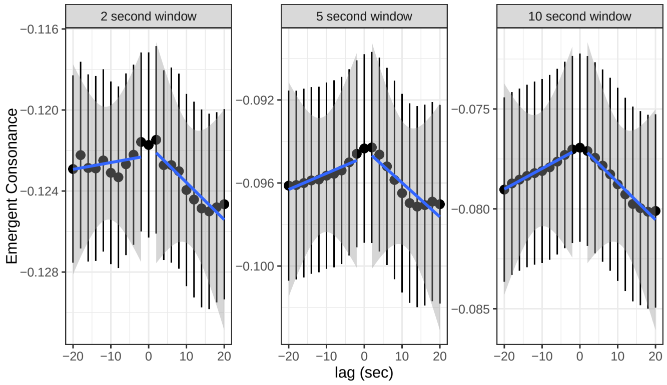

(b) Lagged emergent consonance in coupled trials.

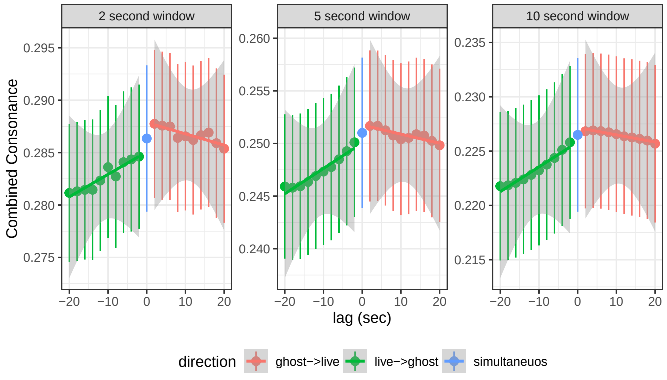

(c) Lagged combined consonance in one-way trials.

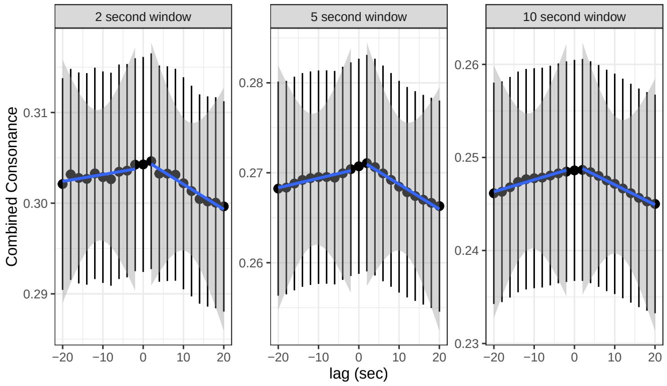

(d) Lagged combined consonance in coupled trials.

Fig. 11. Lagged consonance analysis. Live musicians harmonize with past notes of ghost recording significantly more so than the other way around. This effect is robust across a range of consonance window sizes and was found for both *Emergent* (a) and *Combined* (c) consonance measures.
